# Key miRNAs and Genes in the High-Altitude Adaptation of Tibetan Chickens

**DOI:** 10.3389/fvets.2022.911685

**Published:** 2022-07-14

**Authors:** Binlong Chen, Diyan Li, Bo Ran, Pu Zhang, Tao Wang

**Affiliations:** ^1^College of Animal Science, Xichang University, Xichang, China; ^2^School of Pharmacy, Chengdu University, Chengdu, China; ^3^College of Animal Science and Technology, Sichuan Agricultural University, Chengdu, China

**Keywords:** chicken, miRNAs, genes, high-altitude, adaptation

## Abstract

Tibetan chickens living at high altitudes show specific physiological adaptations to the extreme environmental conditions. However, the regulated base of how chickens adapt to high-altitude habitats remains largely unknown. In this study, we sequenced 96 transcriptomes (including 48 miRNA and 48 mRNA transcriptomes of heart, liver, lung, and brain) and resequenced 12 whole genomes of Tibetan chickens and Peng'xian yellow chickens. We found that several miRNAs show the locally optimal plastic changes that occurred in miRNAs of chickens, such as miR-10c-5p, miR-144-3p, miR-3536, and miR-499-5p. These miRNAs could have effects on early adaption to the high-altitude environment of chickens. In addition, the genes under selection between Tibetan chickens and Peng'xian yellow chickens were mainly related to oxygen transport and oxidative stress. The I-kappa B kinase/NF-kappa B signaling pathway is widely found for high-altitude adaptation in Tibetan chickens. The candidate differentially expressed miRNAs and selected genes identified in this study may be useful in current breeding efforts to develop improved breeds for the highlands.

## Introduction

Because of many malconditions, such as more intensive ultraviolet radiation, lower atmospheric pressure, and lower oxygen partial pressure, the high-altitude plateau is unfavorable to animal survival; however, it is so astounding that many kinds of animals are living there. Some morphological studies indicate that high-altitude species with medium or large builds, such as yak, Tibetan mastiff, and Tibetan pig, have different tissue structures and blood biochemical indexes from their close relative species (1–3). These studies showed that plateau animals usually have more capillaries in the lung, thinner blood-gas barrier, and exceptional abundant red cells and hemoglobin. There can be no doubt that these animal species have adapted to high-altitude environments. Phenotypic plasticity is the capacity that a single genotype can produce different phenotypes in response to environmental change (4). The role of phenotypic plasticity in adaptive evolution is a controversial issue that whether plasticity constrains or facilitates adaptive evolution (5–9). Our previous research showed that phenotypic plasticity could help chickens readapt to their ancestral environments (10). Anyway, plasticity changes are widespread when organisms are faced with new or altered environments.

To investigate whether some genes of these animals are changed in the progression of plateau environmental adaptation, many genome studies have been conducted. The study on domestic yaks (*Bos grunniens*) and their closely related low-altitude cattle (*Bos taurus*) showed that *Adam17, Arg2*, and *Mmp3* are low, especially in strong positive selection in yaks. All three genes are related to hypoxia-inducible factor-1α (11). Another study indicated that some genes that are involved in DNA repair and the production of ATPase are low in positive selection in Tibetan antelope (*Pantholops hodgsonii*) (12). Some genomic studies on pigs (13), chickens (14), and hot-spring snakes (15) also demonstrate similar findings. The results of genomic studies reveal the inherent differences between high-altitude animals and their low-altitude close relative species. Only a small number of studies focus on the regulatory factors of genes, such as microRNA (miRNA). miRNA is one kind of small noncoding RNA with 18–25 nt in length. Many studies have shown that miRNAs widely participate in various biological processes, such as angiogenesis (16, 17), DNA damage repair (18, 19), and erythropoiesis (20, 21). Few studies reveal the miRNA expression profile of high-altitude native animals and their low-altitude close relatives (22, 23). It is still unclear whether miRNA plays extensive roles in high-altitude adaptation.

The Tibetan chicken is one of the chicken breeds found on the Tibetan plateau dating back to the seventh century A.C., which are widely distributed in farming areas of Tibet, including Shigatse, Lhasa, Lhoka, and Nyingchi (24). A previous study showed that incubation at high altitude of fertilized eggs laid by sea-level hens markedly restricted fetal growth compared with those laid by high-altitude hens; in contrast, incubation at sea level of fertilized eggs laid by high-altitude hens not only restored but enhanced fetal growth (25), which suggested that these hens have a high-altitude adaption. The basis of genetic adaptations to the extreme environmental conditions of the Tibetan plateau has recently been partly investigated in Tibetan chickens (26–28). At transcriptome levels, chorioallantoic membrane samples of Tibetan chickens and Chahua chickens were analyzed to explore hypoxic adaptation in Tibetan chickens (29). Comparative transcriptomic and proteomic analyses indicated that differentially expressed genes and proteins were mainly enriched in angiogenesis pathways that might be helpful for hypoxic adaptation in the embryos of Tibetan chickens (30). miR-15a was significantly increased in embryonic lung tissue (31) and GgmiRNA-454 is a time-dependent and tissue-differential expression miRNA of Tibetan chickens (32). Furthermore, several key proteins and pathways were identified and considered important candidates for high-altitude adaptation in Tibetan chickens (33). However, transcriptome analysis, especially of these regulatory RNAs such as miRNA across multiple tissues, environments, and breeds, was rarely conducted to explore the mechanism of high-altitude adaption of the Tibetan chicken populations.

In this study, we performed the reciprocal transplant test (highland and lowland chicken breeds raised in both highland and lowland environments) of Tibetan chickens and a lowland chicken breed (Peng'xian yellow chicken) to explore the miRNAs involved in the process of plateau environment adaptation of Tibetan chickens. In addition, whole-genome resequencing of Tibetan chickens and Peng'xian yellow chickens was also conducted. Based on miRNA and mRNA data from heart, liver, lung, and brain tissues integrated with whole-genome resequencing data, we explored how chickens adapt to high-altitude environments. Our work uncovers several miRNAs have a plastic change as the living environment (altitude) changes. These miRNAs could be involved in the response of hypoxia, inflammation, or other stress under a high-altitude environment and help chickens adapt, indicating that miRNAs could play key roles in the adaptation to a high-altitude environment.

## Materials and Methods

### Chicken Breeds and Sample Collection

Tibetan chickens (the high-altitude chicken breed) were hatched in A'ba (altitude, 3,300 m) and Ya'an (altitude, 670 m), and the same operation was performed on Peng'xian yellow chickens (the low-altitude chicken breed). All the chickens were fed normally. When the experimental chickens were 120 days old, for each group, we collected blood samples and four tissue samples (heart, liver, lung, and brain) from three healthy males with similar body weights. The blood samples and tissue samples were stored at −20°C and −80°C until DNA and RNA extraction. These 12 individuals were genome resequenced (Supplementary Table 1) and 48 tissue samples from them were transcriptome sequenced.

### RNA Isolation and Sequencing

The standard TRIzol method was used to isolate the total RNA from tissue samples (heart, liver, lung, and brain). The concentration and purity of RNA were determined using a Nanodrop ND-2000 spectrophotometer (Thermo Fisher Scientific, Wilmington, DE, USA), and the integrity of RNA was confirmed *via* a 2% agarose gel. Using an Agilent 2100 Bioanalyzer (Agilent, Palo Alto, CA, USA), the RNA integrity number (RIN) value was obtained. Using the QiaQuick PCR Purification Kit, the mRNA library was constructed. From the total RNA, 1 μg of RNA was obtained. By using the Truseq^TM^ Small RNA Sample Preparation Kit, adapter ligation and reverse transcription-polymerase chain reaction (PCR) were performed to obtain the cDNA. Sequencing was performed based on the HiSeq platform (Illumina; San Diego, CA, USA).

### DNA Extraction and Sequencing

Genomic DNA was extracted from blood samples using the traditional phenol-chloroform protocol. DNA purity and quality were assessed using a Nanodrop ND-2000 spectrophotometer (Thermo Fisher Scientific, Wilmington, DE, USA), 2% gel electrophoresis, and an Agilent 2100 Bioanalyzer (Agilent, Palo Alto, CA, USA). The sequencing library was constructed using the TruSeq DNA Sample Preparation Kit (Illumina Inc., San Diego, CA, USA) following the manufacturer's protocols. The whole-genome resequencing was performed based on the HiSeq platform (Illumina; San Diego, CA, USA).

### Analysis of Chicken Transcriptome

**Figure 1:**
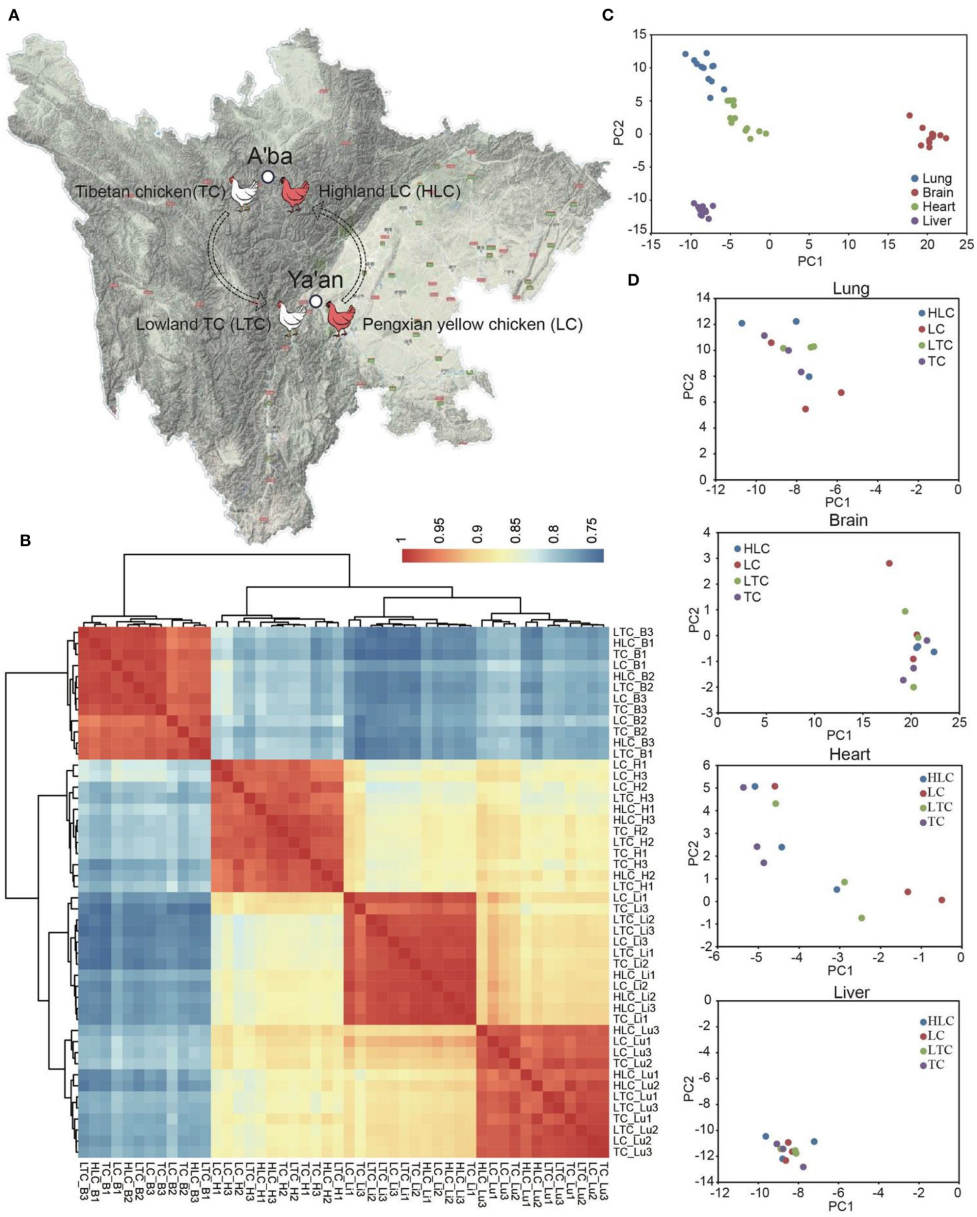
The locations and animal groups of this study. **(A)** Sample collection sites. As the gray deepens, the altitude gets higher. The color of the chicken in this picture does not represent the real color of Tibetan chicken and Peng'xian yellow chicken. **(B)** Heatmap shows the correlation of all samples by Pearson correlation analysis based on CPM (B, H, Li, and Lu represent brain, heart, liver, and lung, respectively). **(C,D)** PCA plots show the difference in all samples and the samples of the same tissue.

To explore the function of miRNA in the process of plateau environment adaptation of lowland chickens, we performed miRNA sequencing of four tissues (heart, liver, lung, and brain) from Peng'xian yellow chickens, which were raised in Ya'an (“LC” hereinafter) and A'ba (“HLC” hereinafter), and Tibetan chickens, which were raised in Ya'an (“LTC” hereinafter) and A'ba (“TC” hereinafter) (Figure 1A).

For miRNA data, Cutadapt version 1.12 (34) was used to remove the adaptor sequences. Trimmed reads were compared to known miRNAs of chicken from the miRbase database (version 22) by Bowtie (35). Next, after filtering known miRNA sequences, the remaining sequences were BLAST searched against the *Gallus gallus* genome. The sequences matching the chicken genome were used to predict the novel miRNA by mirDeep2 (36) using default parameters.

For transcriptome data, we employed TopHat2 (37) to do reads mapping against the chicken reference genome (Ensemble release 92). The miRNA expression level was normalized by edgeR (38). Then, we did correlation analysis (Pearson correlation) and principal component analysis (PCA) by SAS based on count-per-million (CPM), which was generated from edgeR. To identify differentially expressed miRNAs (DEMs), we first did a pairwise comparison by edgeR and obtained six compared groups, namely, “HLC-LC,” “HLC-TC,” “HLC-LTC,” “LC-TC,” “LC-LTC,” and “TC-LTC,” respectively. DEMs were identified with a log2 fold threshold of 1.5, log2 CPM > 2, and FDR ≤ 0.05.

We speculated that due to long-term living in highlands or lowlands, some miRNAs that are related to environmental adaptation have their unique expression patterns in Tibetan chickens and Peng'xian yellow chickens and help chickens adapt to their living environments. In short, that is what we called ENMs. To screen out ENMs, we first identified differentially expressed miRNAs (DEMs) between highland and lowland experimental chickens. In the samples of the same tissue, known miRNAs were compared in pairs to identify DEMs using edgeR. We focused on the miRNAs that are caused by the EN factor. So, we merged the DEMs that are in every comparative group of the same tissue to reduce the influence of other factors. The concrete implementation method is as follows.

In the comparative groups of the same tissue, the EN factor works in four groups (“TC-LC, “TC-LTC,” “HLC-LC,” and “HLC-LTC,” respectively) that we named effective groups. Because of the same growth environment, the EN factor does not exist in the other groups (“TC-HLC” and “LTC-LC”) that we named ineffective groups. In consideration of the condition that ENMs should be stable in effective groups, we focused on the miRNAs that are at the intersection of the effective groups and not in the ineffective groups.

We then screened miRNAs that changed with the environment to evaluate whether the optimal plasticity changes of miRNAs occurred in the chickens of the “HLC” group. In this study, the differentially expressed miRNAs can be divided into three parts, namely, caused by breed (BR), environment (EN), and experiment error (EE), respectively. For example,

DEMs TC-LC = FBR + FEN + FEE. In this group, there are different chicken breeds and different areas where these chickens grew up. So, the differentially expressed miRNAs were caused by all three factors in the “TC-LC” group.

DEMs HLC-TC = FBR + FEE. In this group, both Peng'xian yellow chickens and Tibetan chickens grew up in the same environment, A'ba. There is no environmental factor.

DEMs HTC-LTC = FEN + FEE. In this group, Tibetan chickens were raised in different areas, A'ba and Ya'an. BR factor was not working here.

To investigate the role of ENMs, we employed miRDB to predict their target genes. To ensure the reliability of the prediction results, we only keep the part the target score >80 in miRDB. Functional enrichment analysis of GO terms, KEGG pathway analysis, and pathway enrichment were performed using Metascape. Because there is no chicken database in Metascape, we map the genes to human homologous genes for further analysis.

### Chicken Genome Resequencing Analysis

Trimmomatic (39) was used to remove adapter and low-quality data. Trimmed reads were compared to the chicken reference genome (GRCg6a) by Burrows-Wheeler Aligner (BWA). SAMtools (40) and GATK (41) were used to detect single nucleotide polymorphisms (SNPs), and the software Annovar (42) was used to annotate the SNPs with genomic elements. Subsequently, we did a selective sweep analysis. A 40-kb sliding window and a 20-kb step size were used to detect the selective sweep region of the genome. PopGenome (43) was used to calculate the *Fst* and π ratio of the SNPs in each window. Significantly, GO terms and KEGG pathways of the gene with *Fst* ≥ 0.3 were identified using Metascape (44) with *P* ≤ 0.01.

## Results

### Summary of the miRNA and mRNA Data

In this study, miRNA data were analyzed using Illumina deep sequencing technology. A total of 508.8 million clean reads were obtained. After annotation, a total of 1,079 known miRNAs were identified in all the samples. In total, 269.15 Gb of raw data from 48 mRNA libraries were obtained, with an average of 5.51Gb per sample. After quality filtering, 264.36 Gb of clean data with an average of 94.85% of Q20 was retained for further analysis.

To evaluate the correlation of samples in this study, we calculated the correlation coefficient between every two samples using the Pearson correlation method. The figure showed that the samples of the same tissue have higher correlations than the samples of different tissues for both miRNA and mRNA (Figure 1B). There is a very high correlation between every two samples of the same tissue, which is not subject to breed or growth environments. It seems like altitude has a small effect on the miRNA transcriptome of the chicken. Furthermore, we used the principal component analysis (PCA) method to reduce the dimensionality of these data and further assess the difference in samples. The result of PCA showed similar characteristics to correlation analysis. The difference between the different tissue samples is obvious (Figure 1C). The first three eigenvalues can explain the sample variability of 36.17% (PC1), 15.28% (PC2), and 11.26% (PC3), respectively. But the arrangement of the same tissue samples is chaotic (Figure 1D).

### miRNAs Showed the Locally Optimal Plasticity Changes Alongside Altitude Change

In this study, a total of 1,076 known miRNAs were detected. In addition, 354 novel miRNAs were detected (Supplementary Table 2). For these known miRNAs, 45 DEMs were detected. A total of 22, 21, 7, and 1 DEMs were detected in the liver, lung, heart, and brain, respectively.

In this study, the influence of the environmental (EN) factor is mainly reflected in the change in altitude. We focused on the miRNAs that are caused by the EN factor. The expression of miRNAs in this part is characterized by the change in higher elevation. It showed the plasticity of miRNAs. These miRNAs may play a key regulatory role in the process of plateau environmental adaptation of chickens. We merged these groups that include the EN factor to screen out the miRNAs that are caused by the EN factor. We focused on the miRNAs that always have a high expression level in the experimental chickens at a certain altitude, regardless of the chicken breeds. We named them EN miRNAs (ENMs).

**Figure 2:**
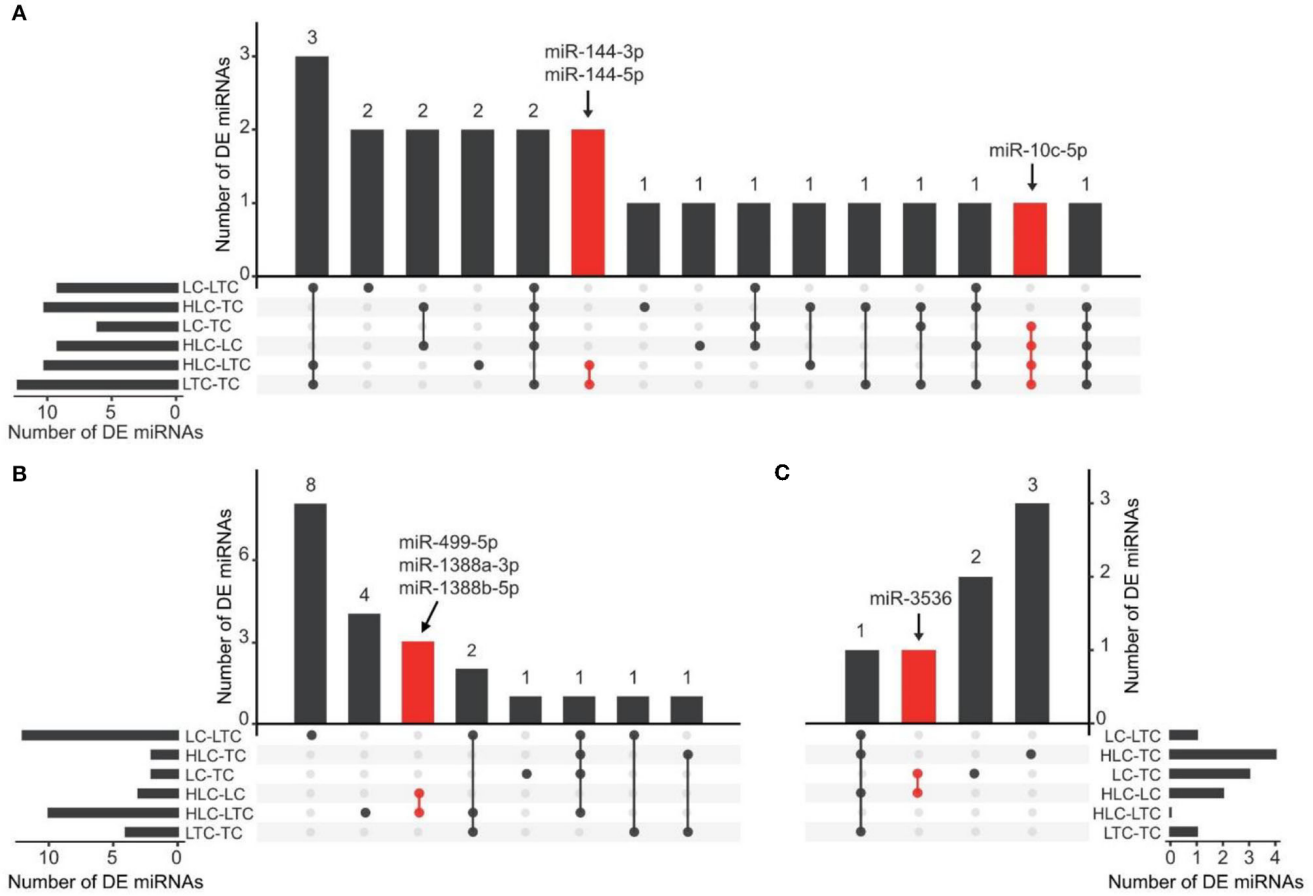
UpSet plot showing the screening of candidate ENMs in the liver **(A)**, lung **(B)**, and heart **(C)**. The bars on the left or right of each panel were the numbers of DE miRNAs in each comparison of two groups of chickens. The dots represent relative DE miRNAs detected in the corresponding comparison. For example, in **(B)**, the red dots represent miRNAs detected to be differentially expressed in both two comparisons (HLC vs. LC, and HLC vs. LTC). Three and two candidate ENMs were obtained from the LC vs. HLC and LTC vs. TC comparisons, respectively.

In the liver, nine miRNAs have obvious expression changes in experimental chickens at high and low altitude environments. They are miR-1692, miR-10c-5p, miR-10b-5p, miR-144-5p, miR-144-3p, miR-2184-5p, miR-375, miR-1736-3p, and miR-205a, respectively. Six of these nine miRNAs (miR-1692, miR-10b-5p, miR-2184-5p, miR-375, miR-1736-3p, and miR-205a) appeared in the ineffective groups (“HLC-TC” and “LTC-LC”). Among the remaining three miRNAs, miR-10c-5p was always highly expressed in the liver of chickens that grew up in low-altitude areas (“LC” and “LTC”). Conversely, miR-144-5p and miR-144-3p were highly expressed in high-altitude experimental chickens (“TC” and “HLC”). These 3 miRNAs are found at the intersection of four effective groups and were marked (Figure 2) as ENMs.

In the same way, we also checked the intersection of the other tissues. At heart, we only found four DEMs in the effective groups. Two DEMs were found at the intersection of effective groups. In them, miR-375 did not show an obvious altitude preference. miR-3536 was highly expressed in high-altitude experimental chickens. In addition, miR-3536 was not the DEMs of ineffective groups. In lung, six DEMs (miR-122-5p, miR-215-5p, miR-449d-5p, miR-449-5p, miR-1388b-5p, and miR-1388a-3p) were found at the intersection of effective groups. In them, miR-499-5p, miR-1388b-5p, and miR-1388a-3p were highly expressed in a certain living environment. And, these miRNAs did not appear in the DEMs of ineffective groups. We were surprised by the analysis result of the comparative groups of the brain. There was only one DEM (miR-194) in the effective groups and it was not at the intersection.

**Figure 3:**
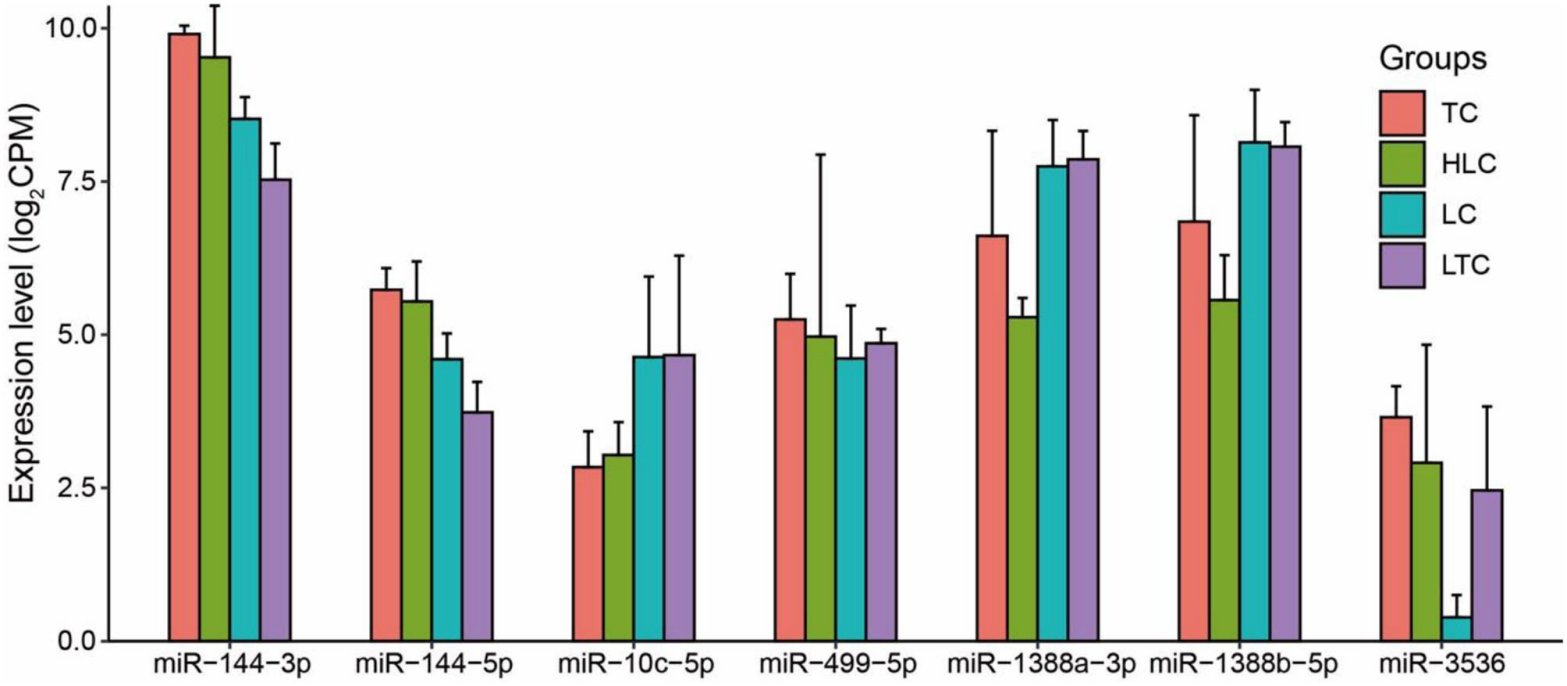
The expression level of candidate miRNAs.

In brief, seven candidate ENMs were obtained from four tissues of experimental chickens. All these screened miRNAs showed plasticity changes in the environment. The expression pattern of these miRNAs was similar to native chickens' when Tibetan chickens and Peng'xian yellow chickens were brought into each other's living environment (Figure 3).

We further used a two-way analysis of variance to further analyze the influence of BR and EN factors on the expression levels of candidate miRNAs (Supplementary Table 3). The results showed that the expression level of all candidate miRNAs was not significantly affected by genetic factors, and the interaction effect of BR and EN factors was significantly affected by the expression of some miRNAs, such as miR-144-3p, miR-144-5p, miR-1388a-3p, and miR-3536. The expression levels of miR-10c-5p and miR-1388b-5p were only significantly affected by EN factors. Their expression levels in low-altitude chicken groups (LC and LTC) were significantly higher than those of high-altitude chicken groups (TC and HLC). miR-10c-5p and miR-1388b-5p showed the plasticity changes when altitude changed and were defined as ENMs. Then we explored the function of ENMs in the altitude adaption of chickens.

### Function Prediction of Environment-Related miRNAs

**Figure 4:**
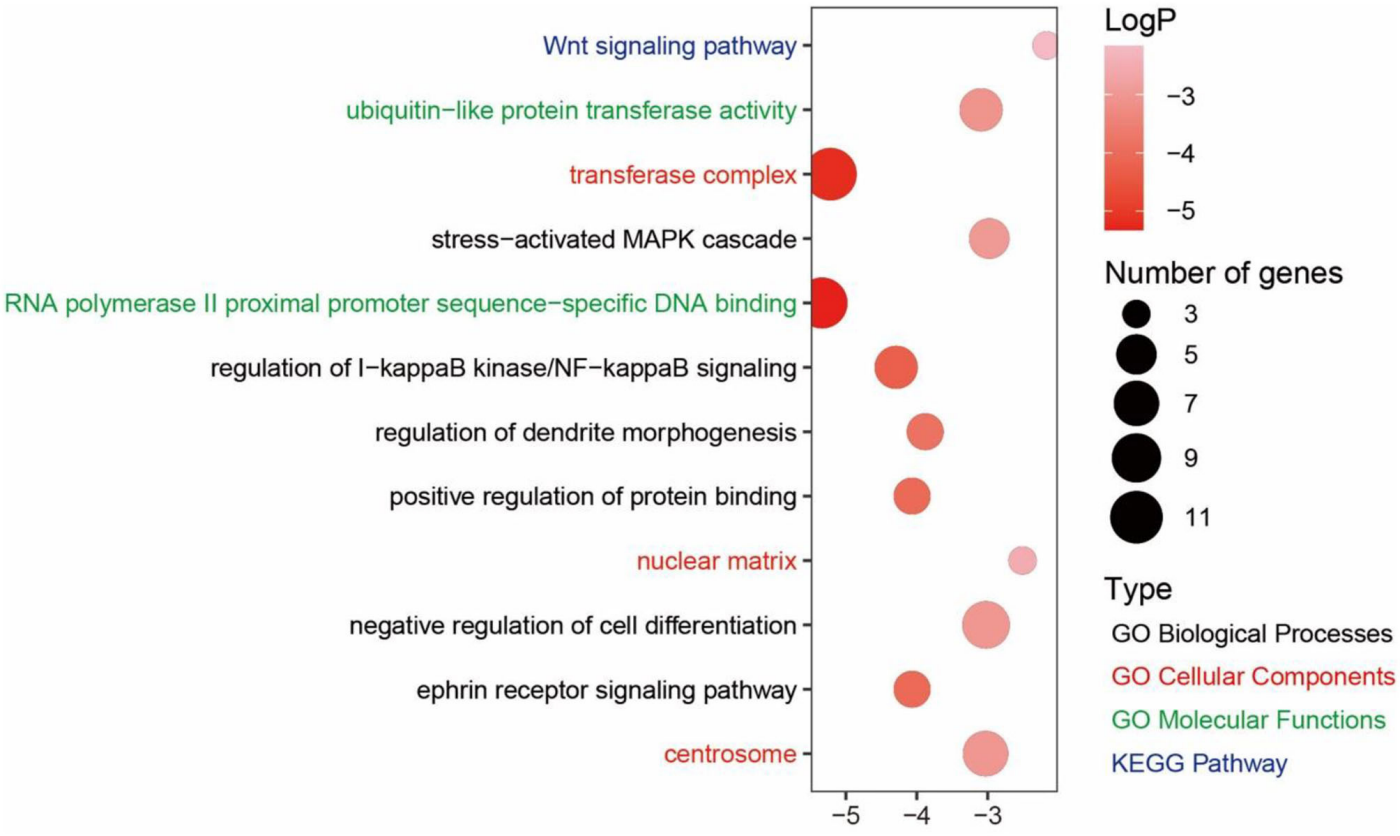
Enrichment analysis of miR-10c-5p.

We combined miRDB and Metascape to explore the functions of environment-related miRNAs (ENMs). miRDB was used to predict the target gene of ENMs. For miR-10c-5p and miR-1388b-5p, we found a total of 82 target genes, 67 and 15, respectively. We then performed enrichment analysis of these target genes using Metascape. The target genes of miR-10c-5p were significantly enriched in a total of 73 GO terms and 3 KEGG pathways (*P* ≤ The target genes of miR-10c-5p were significantly enriched in a total of 73 GO terms The target genes of miR-10c-5p were involved in the GO terms related to high-altitude stress, such as “regulation of I-kappaB kinase/NF-kappaB signaling” (GO:0043122), “ephrin receptor signaling pathway” (GO:0048013), and “stress-activated MAPK cascade” (GO:0051403) (Figure 4). In addition, the target genes of miR-1388b-5p were significantly enriched in 7 GO terms and 0 KEGG pathways, such as “protein serine/threonine kinase activity” (GO:0004674) and “maintenance of location” (GO:0051235) (Supplementary Figure 1).

Studies have shown that increased exposure to hypoxia could cause insufficient oxygen supply for tissue and further induce inflammation (45, 46). As mentioned above, we found that some target genes of miR-10c-5p are involved in the “regulation of I-kappaB kinase/NF-kappaB signaling” (GO:0043122) and “ephrin receptor signaling pathway” (GO:0048013). It showed that miR-10c-5p may help chickens resist the hypoxic environment.

### The Target Genes of miR-10c-5p Showed Locally Optimal Plasticity Changes When the Environment Changed

To evaluate differentially expressed mRNAs (DEGs) between different tissues, breeds, and environments, we compared the expression levels with the threshold of FC (fold change) ≥ 2 (or ≤ 0.5) plus a Bonferroni-adjusted *P* adjusted a Boa t-test. Overall, we compared 28 pairs and identified 18–1,634 mRNAs as significantly differentially expressed (Supplementary Table 4). These DEGs were involved in 0–97 pathways (Supplementary Table 4). The genes highly expressed in Tibetan chickens were found to be enriched in immune-related GO terms (such as “innate immune response,” “defense response to virus,” and “cytokine activity”) (Supplementary Figure 2).

**Figure 5:**
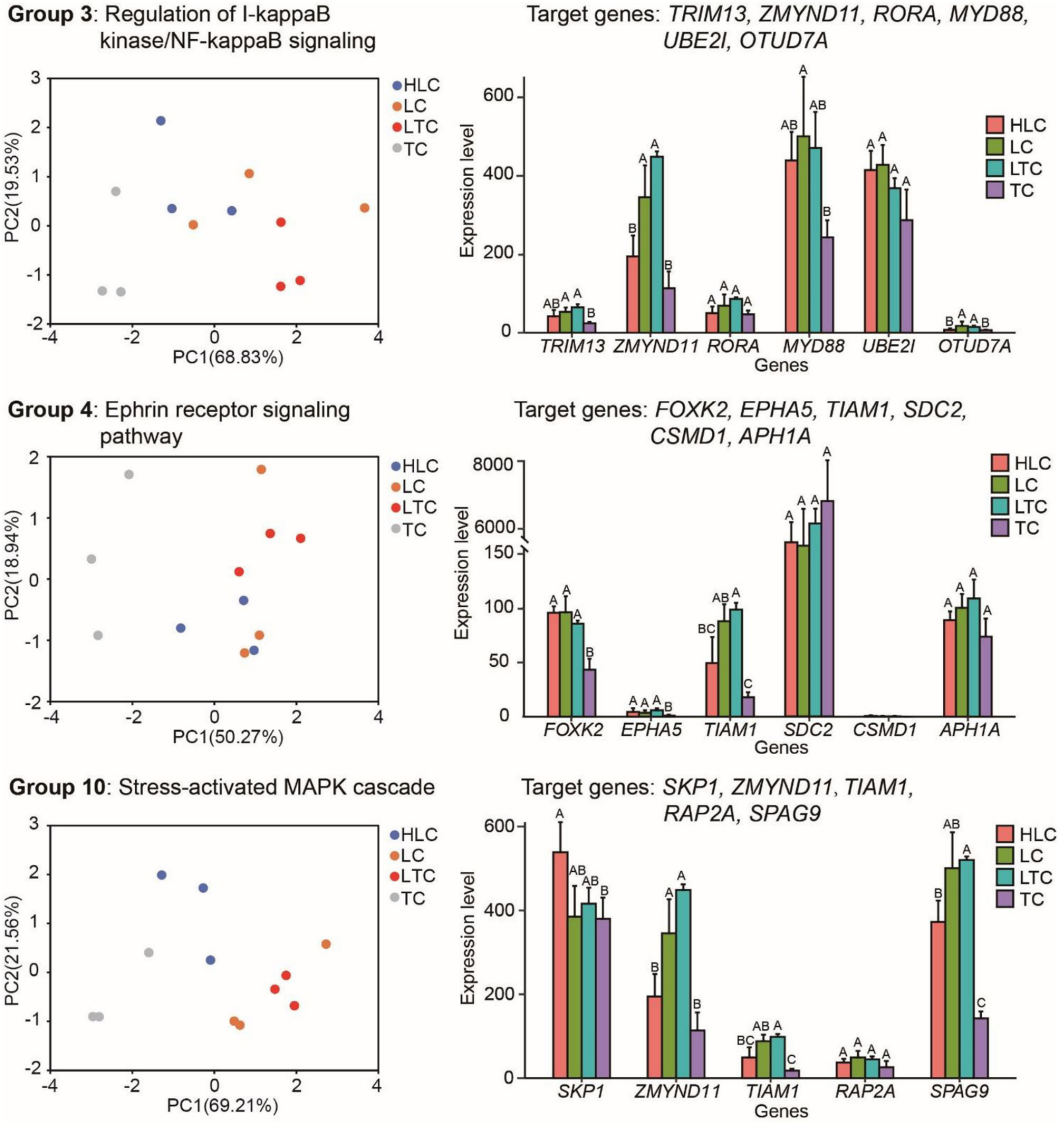
Target genes of miR-10c-5p have different expression patterns between high and low-altitude chickens. The left, middle, and right panels represent the target genes of miR-10c-5p and the pathways these genes are involved in (left panels), PCA plots based on these target genes, and the expression of target genes of miR-10c-5p, respectively. Different letters mean that there is a statistically significant difference between groups (*P* ≤ 0.05).

miR-10c-5p shows a strong altitude preference. Its target genes may also show functional adaptations when the living environment is changed. Then we explored the expression pattern of the target genes of miR-10c-5p. In fact, through joint analysis of miRNA data and transcriptome data, we found that expression pattern change occurred in several genes that are involved in these GO terms between high and low-altitude experimental chickens (Figure 5), such as zinc finger MYND-type containing 11 (*ZMYND11*), OTU deubiquitinase 7A (*OTUD7A*), tripartite motif-containing 13 (*TRIM13*), and T-cell lymphoma invasion and metastasis 1 (*TIAM1*). *ZMYND11* has a lower expression level in high-altitude experimental chickens (“HLC” and “TC” groups) than in low-altitude chickens (“LTC” and “LC” groups). *OTUD7A, TRIM13*, and *TIAM1* have similar expression patterns. As shown in Figure 5, both the PCA plot and histogram showed that there is no significant difference between lowland chicken groups (“LC” and “LTC”) in these genes. Analogously, the expression pattern of these genes in chickens of the “HLC” group was similar to those of the “TC” group. It indicated that some target genes of miR-10c-5p also showed optimal plasticity changes when the environment changes.

### The Genes That Are Under Selection Were Related to Oxygen Transport and Oxidative Stress

We obtained a total of 235.65 Gb of clean reads from genome resequencing. After mapping to the genome, the reads with unique read alignments were used to analyze common genetic polymorphisms, whereas the remaining reads were discarded. In all, 10,819,586 SNPs were identified from all chickens, of which 150,897 were from the exonic region (Supplementary Table 5).

**Figure 6:**
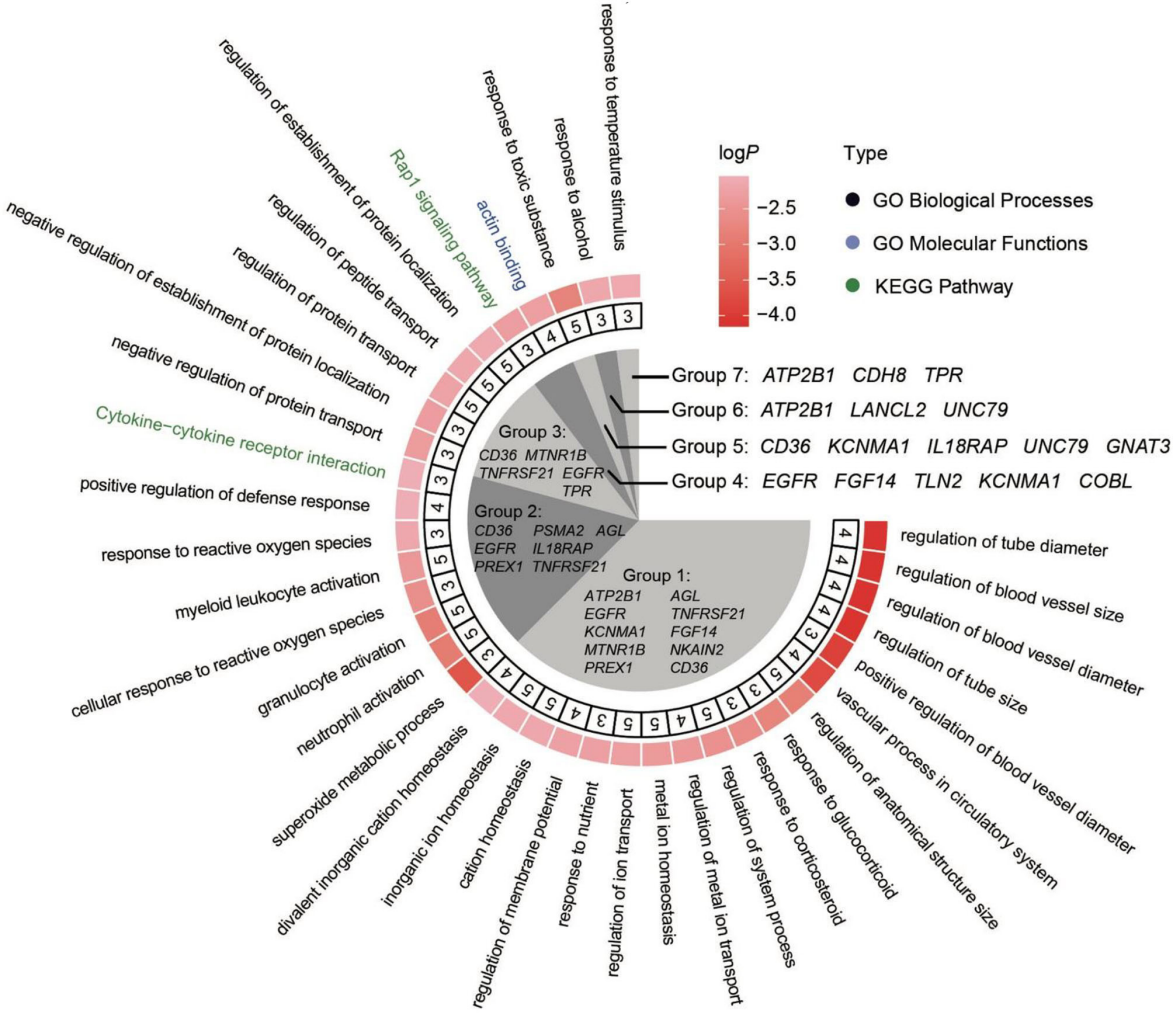
Enrichment analysis of the genes under selection. All enrichment terms can be divided into seven groups. The genes involved in these groups were marked in the inner fan. The second annulus represents the number of genes that are involved in the specific terms.

To detect the regions with extreme divergence in allele frequency (*Fst*) on autosomes, we scanned the genome regions in 40-kb sliding windows. In total, we identified 82 candidate regions (with Fst ≥ 0.3) that included 50 genes. We further investigated the function of these genes. Results showed that the top-ranked gene ontology (GO) terms were related to vasculature development, including regulation of blood vessel size, regulation of blood vessel diameter, regulation of tube size, and vascular process in the circulatory system. Comparisons against the Kyoto Encyclopedia of Genes and Genomes (KEGG) indicated that several genes were clustered into cytokine–cytokine receptor interaction and Rap1 signaling pathway (Figure 6).

**Figure 7:**
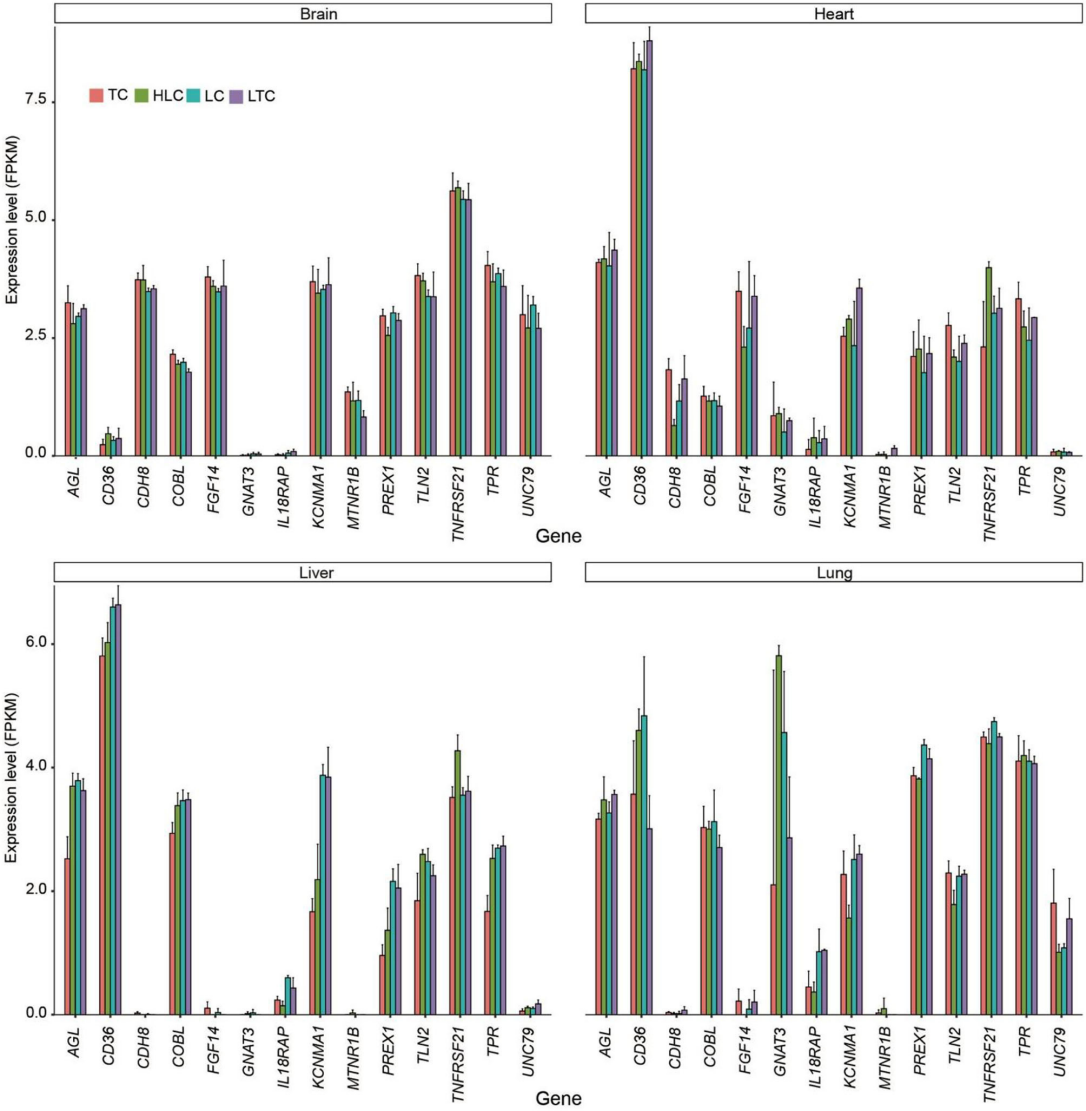
The expression level (log_2_FPKM) of selected genes in four tissues.

Combined with transcriptome results, we further analyzed the expression of selected genes in multiple tissues (Figure 7). Among these genes, *ATP2B1, EGFR, KCNMA1, MTNR1B*, and *PREX1* were associated with the regulation of anatomical structure size, such as blood vessel diameter.

## Discussion

The Tibetan chicken is a unique breed that has adapted to the high-altitude hypoxic conditions of the Tibetan plateau (30). A number of positively selected genes have been reported in these chickens; however, the mechanisms of gene expression and regulation for hypoxia adaptation are not fully understood. In this study, we conducted reciprocal transplant experiments of Tibetan chicken and Peng'xian yellow chicken to investigate whether some miRNAs have a plastic response to a different environment and play roles in the progress of plateau environment adaptation of chickens. Furthermore, through genome analysis, we found that the genes that are related to the regulation of blood vessel diameter and cellular response to reactive oxygen genes are under selection between Tibetan chicken and Peng'xian yellow chicken. Some selected genes showed altitude-specific expression, and it indicated that these genes are under regulation.

At the miRNA transcriptome level, we found more differentially expressed miRNAs (DEMs) in liver and lung samples than in the heart and brain, which indicated that the functions of the liver and lung could play an important role in the progress of chickens' plateau environmental adaptation. This is similar to other mammals living on the Tibetan plateau. The low-altitude cattle (*Bos taurus*) are closely related to domestic yak (*Bos grunniens*). The divergence time for them is five million years ago (11). The research on yaks and cattle found a total of 85 differentially expressed mature miRNAs in the lung (70 DEMs) and heart (29 DEMs) with a threshold fold change > 2 and FDR < 0.05 (47). The research on Tibetan pigs and Yorkshire pigs found 51 DEMs in the liver with the threshold of Fisher's exact test *P* < 0.01 and fold change > 2 (48). These results suggest that neural activity is very sensitive to the change in oxygen concentration. When the body is in a low-oxygen environment, the oxygen may be preferentially used for brain supply, resulting in a sufficient oxygen supply for the brain.

Correlation analysis and PCA showed a high correlation and low difference between every two samples in the same tissue. In fact, we only found a few DEMs between every two groups. Through the selection of DEMs of all comparative groups, we observed that some miRNAs have a significant environmental preference. Further analysis revealed that, although Tibetan chickens adapt to the highland in many ways, it shows similar characteristics as lowland chickens in miRNA and transcriptome when Tibetan chickens are brought back to the lowland (10). Some miRNAs and genes of Peng'xian yellow chickens also show plastic changes when they grow up in the highland. These miRNAs and genes show a similarity expression pattern with highland chickens. In short, our findings indicated that several miRNAs could play a key role in the process of plateau environmental adaptation of chickens, such as miR-10c-5p and miR-1388b-5p.

It is known that hypoxia can induce the expression of inflammatory cytokines and chemokines (49, 50). The liver, as an immune organ, plays an important role in the hepatic inflammatory response (51). The vast majority of hepatic T cells (Th1/Tc1) secrete inflammatory cytokines, including interferon-γ, TNF-α, and interleukin-2 (52). In our study, miR-10c-5p may play an important role in regulating inflammatory responses in the liver. It always has a high expression level in the livers of lowland experimental chickens, whether Tibetan chickens or Peng'xian yellow chickens. Functional enrichment analysis and pathways analysis showed that some target genes of miR-10c-5p were involved in the “regulation of I-kappaB kinase/NF-kappaB signaling,” “ephrin receptor signaling pathway,” and “stress-activated MAPK cascade.” A recent study also indicates that genes related to Tibetan chicken environmental adaptations were involved in the “regulation of I-kappaB kinase/NF-kappaB signaling” (24) and MAPK (29) pathways. Differentially expressed miRNAs analysis also found the targeted genes were involved in the I-kappa B kinase/NF-kappa B signaling pathway (53). These previous and current studies suggest the key role of the I-kappa B kinase/NF-kappa B signaling pathway in the hypoxia adaptation of Tibetan chickens.

At the mRNA transcriptome level, further analysis of miR-10c-5p's target genes that were involved in the enrichment groups as described above found that four genes, namely, *ZMYND11, OTUD7A, TRIM13*, and *TIAM1*, were expressed differentially between lowland and highland experimental chickens. These genes have significantly lower expression in the TC group than in the lowland experimental groups (“LTC” and “LC”). While in the HLC group, the expression level of these genes is biased to the TC group. It indicates that these genes could play an important role in the progress of high-altitude adaptation of chickens, such as *ZMYND11*. *ZMYND11*, and zinc finger MYND-type containing 11, is known as *BS69* and works as a transcriptional repressor (54, 55). Research shows that *ZMYND11* could negatively regulate the NF-kappaB signaling pathway in chickens (56). In this study, we found that *ZMYND11* has a lower expression level in high-altitude experimental chickens. It indicates that miR-10c-5p may activate the NF-κB signaling pathway by regulating *ZMYND11* in high-altitude chickens' livers, further promoting pro-inflammatory cytokine expression to defend against the tissue inflammation that is caused by hypoxia at highland. The specific roles of these genes in the form of high-altitude adaptation need more studies to determine.

At the genome level, a total of 50 candidate genes were identified using a sliding window analysis. Hypoxia typically causes a release of intracellular Ca^2+^, mediated by the ryanodine receptors, which then leads to increased cell contraction (57). Consistent with previous studies, which indicated that several candidate genes in the calcium-signaling pathway are possibly involved in adaptation to the hypoxia experienced by Tibetan chickens (26). In this study, *ATP2B1* (ATPase plasma membrane Ca2+ transporting 1) plays a key role in intracellular calcium homeostasis and is further involved in vascular smooth muscle contraction and regulation of blood pressure (58, 59), and was also identified to be potentially positively selected. *EGFR* (epidermal growth factor receptor) is the receptor for EGF, which is involved in the mitogenic, stimulating proliferation of many cell types, including human microvascular endothelial cells (60) and lymphatic endothelial cells (61). *PREX1* (phosphatidylinositol-3,4,5-trisphosphate-dependent Rac exchange factor 1) is a member of the Rac exchange factor family of guanine nucleotide exchange factors. The *PREX* proteins can activate Rho GTPases, which regulate cell motility, proliferation, glucose uptake, and reactive oxygen species generation (62). Some selected genes showed environment-specific expressions, such as *KCNMA1* and *PREX1*. In the lung, the expression levels of these genes were similar in low-altitude chickens (“LC” and “LTC”) and similar in high-altitude chickens (“TC” and “HLC”). It showed that the expression of these genes is under the action of regulatory factors.

## Conclusion

In summary, our study uncovers that several miRNAs have a plastic change alongside living environment (altitude) changes. These miRNAs could be involved in the response to hypoxia, inflammation, or other stresses in high-altitude environments and help chickens adapt. The target genes of these miRNAs also showed differential expression under different environmental conditions. Further genome analyses revealed that several candidate genes in the calcium-signaling and immunity pathways are possibly involved in adaptation to the hypoxia experienced by these Tibetan chickens. The I-kappa B kinase/NF-kappa B signaling pathway was widely found in the hypoxia adaptation of Tibetan chickens. The candidate differentially expressed miRNAs, genes, and selected genes identified in this study may be useful targets for breeding efforts to develop improved chicken breeds for the Tibet plateau.

## Data Availability Statement

The datasets presented in this study can be found in online repositories. The names of the repository/repositories and accession number(s) can be found in the article/Supplementary Material.

## Ethics Statement

The animal study was reviewed and approved by Institutional Animal Care and Use Committee at the Chengdu University.

## Author Contributions

DL and TW conceived the study and the analytical strategy. BC wrote the manuscript. BR and PZ performed the statistical analyses. TW revised the article. All authors have read and agreed to the published version of the manuscript.

## Funding

This study was supported by Sichuan Science and Technology Program Grants 2019JDTD0009, 2020YFH0138, and 2020YFSY0040.

## Conflict of Interest

The authors declare that the research was conducted in the absence of any commercial or financial relationships that could be construed as a potential conflict of interest.

## Publisher's Note

All claims expressed in this article are solely those of the authors and do not necessarily represent those of their affiliated organizations, or those of the publisher, the editors and the reviewers. Any product that may be evaluated in this article, or claim that may be made by its manufacturer, is not guaranteed or endorsed by the publisher.
