# Supplementary material for: Key miRNAs and Genes in the High-Altitude Adaptation of Tibetan Chickens
Source: Front Vet Sci. 2022 Jul 14;9:911685. doi: 10.3389/fvets.2022.911685 (PMC9330022; doi:10.3389/fvets.2022.911685)
Supplement: Supplementary file 1 [file Data_Sheet_1.docx]

**Supplementary Figures**

**
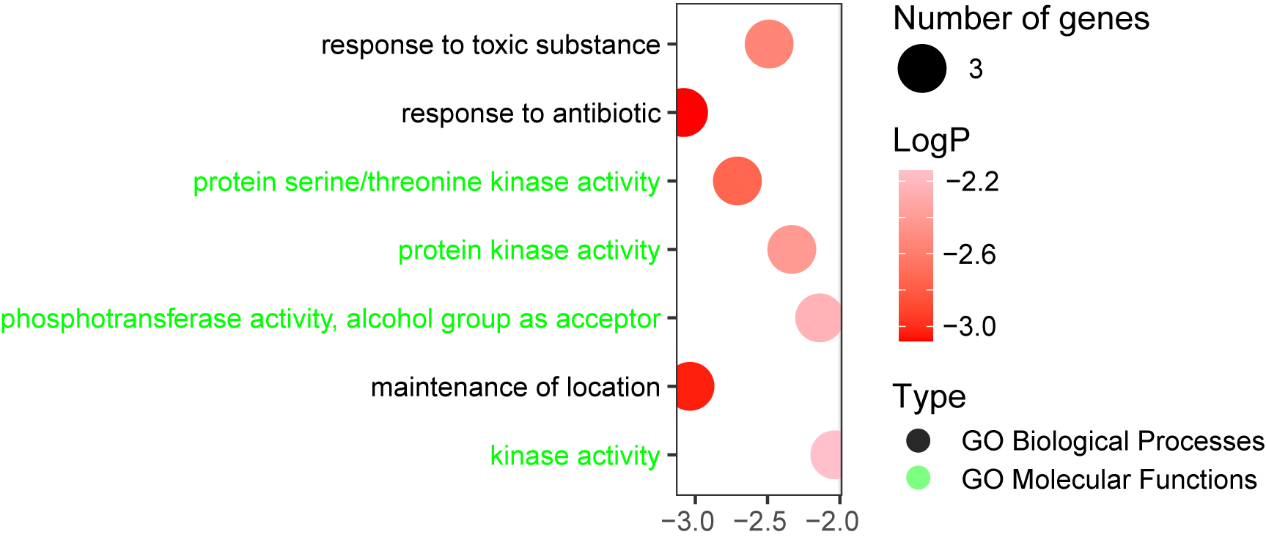
**

**Figure S1.** Enrichment analysis of miR-10c-5p.


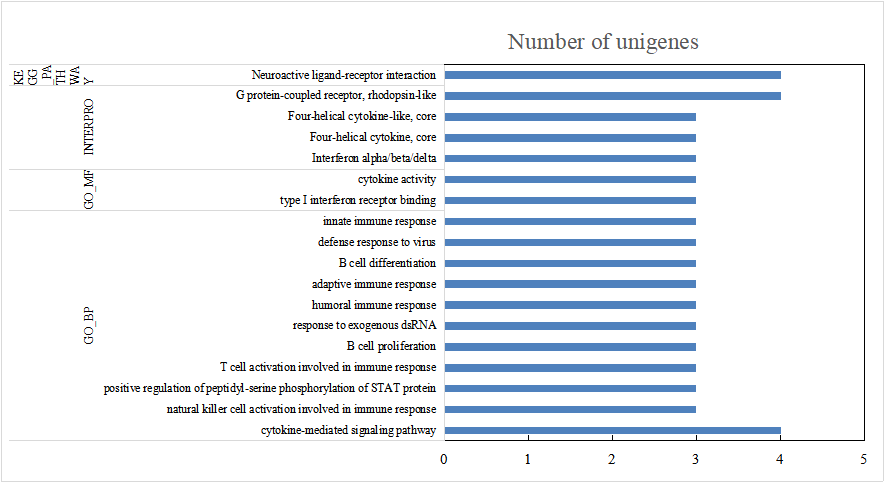


**Figure S2** Functional enrichment analysis of Tibetan chickens relative to lowland chicken specific expression genes.

**Supplementary Tables**

**Table S1** The summary of genome resequencing.

| Breed | Sample name | Clean reads | Mapped reads | Mapped bases (bp) | Mapping ratio (%) | Average depth |
| --- | --- | --- | --- | --- | --- | --- |
| Peng’xian yellow chicken | LC1 | 123,514,556 | 121,549,598 | 17,835,637,408 | 98.41% | 14.54 |
|  | LC2 | 129,943,634 | 127,075,609 | 18,621,923,571 | 97.79% | 14.94 |
|  | LC3 | 121,933,950 | 120,195,056 | 17,634,953,858 | 98.57% | 14.27 |
|  | LC4 | 114,805,466 | 113,134,152 | 16,568,836,970 | 98.54% | 13.70 |
|  | LC5 | 133,732,974 | 131,762,298 | 19,334,435,705 | 98.53% | 15.82 |
|  | LC6 | 124,830,814 | 123,088,068 | 18,072,641,788 | 98.60% | 14.82 |
| Tibetan chicken | TC1 | 132,489,660 | 130,214,243 | 19,091,543,906 | 98.28% | 15.36 |
|  | TC2 | 137,262,560 | 135,246,978 | 19,842,296,951 | 98.53% | 16.02 |
|  | TC3 | 114,718,642 | 112,837,400 | 16,549,336,369 | 98.36% | 13.53 |
|  | TC4 | 155,957,666 | 153,644,772 | 22,545,959,421 | 98.52% | 16.61 |
|  | TC5 | 134,070,044 | 132,104,611 | 19,384,276,513 | 98.53% | 15.78 |
|  | TC6 | 142,092,608 | 140,167,508 | 20,557,962,599 | 98.65% | 16.38 |

**Table S3.** The impact of environment and breed factors on the expression level of candidate miRNAs. Two-Way ANOVA were used to calculate the difference based on log_2_CPM with the tukey method. The significant level is 0.05. PXC and TC represents Peng’xian yellow chicken and Tibetan chicken, respectively.

| Factor | miRNA | Class | Mean | Std | significance |
| --- | --- | --- | --- | --- | --- |
| Environment | miR-144-5p | Low altitude | 4.1664 | 0.6292 | B |
|  |  | High altitude | 5.6379 | 0.4804 | A |
|  | miR-144-3p | Low altitude | 8.0245 | 0.6978 | B |
|  |  | High altitude | 9.7156 | 0.5769 | A |
|  | miR-10c-5p | Low altitude | 4.6516 | 1.3207 | A |
|  |  | High altitude | 2.9379 | 0.5135 | B |
|  | miR-499-5p | Low altitude | 4.6943 | 0.5979 | A |
|  |  | High altitude | 5.0538 | 1.9890 | A |
|  | gga-miR-1388a-3p | Low altitude | 7.7985 | 0.5666 | A |
|  |  | High altitude | 5.9273 | 1.3345 | B |
|  | gga-miR-1388b-5p | Low altitude | 8.1000 | 0.6010 | A |
|  |  | High altitude | 6.1839 | 1.3985 | B |
|  | gga-miR-3536 | Low altitude | 0.6302 | 2.0576 | B |
|  |  | High altitude | 3.0816 | 1.5962 | A |

| Breed | miR-144-5p | PXC | 5.0717 | 0.7123 | A |
| --- | --- | --- | --- | --- | --- |
|  |  | TC | 4.7326 | 1.1617 | A |
|  | miR-144-3p | PXC | 9.0235 | 0.7976 | A |
|  |  | TC | 8.7166 | 1.3575 | A |
|  | miR-10c-5p | PXC | 3.8357 | 1.2542 | A |
|  |  | TC | 3.7538 | 1.4808 | A |
|  | miR-499-5p | PXC | 4.7269 | 2.0095 | A |
|  |  | TC | 5.0212 | 0.5487 | A |
|  | gga_miR_1388a_3p | PXC | 6.4998 | 1.4548 | A |
|  |  | TC | 7.2261 | 1.3303 | A |
|  | gga_miR_1388b_5p | PXC | 6.8366 | 1.5933 | A |
|  |  | TC | 7.4472 | 1.3258 | A |
|  | gga_miR_3536 | PXC | 0.8523 | 2.4799 | A |
|  |  | TC | 2.8595 | 1.3490 | A |

| Environment  * Breed | miR-144-5p | PXC | Low altitude | 4.6005 | 0.4199 | AB |
| --- | --- | --- | --- | --- | --- | --- |
|  |  | PXC | High altitude | 5.5429 | 0.6529 | A |
|  |  | TC | Low altitude | 3.7323 | 0.4981 | B |
|  |  | TC | High altitude | 5.733 | 0.3515 | A |
|  | miR-144-3p | PXC | Low altitude | 8.521 | 0.3561 | AB |
|  |  | PXC | High altitude | 9.5261 | 0.8403 | A |
|  |  | TC | Low altitude | 7.528 | 0.5923 | B |
|  |  | TC | High altitude | 9.9052 | 0.1353 | A |
|  | miR-10c-5p | PXC | Low altitude | 4.635 | 1.3145 | A |
|  |  | PXC | High altitude | 3.0363 | 0.5366 | A |
|  |  | TC | Low altitude | 4.6681 | 1.6223 | A |
|  |  | TC | High altitude | 2.8395 | 0.585 | A |
|  | miR-499-5p | PXC | Low altitude | 4.5644 | 0.8867 | A |
|  |  | PXC | High altitude | 4.8894 | 3.0380 | A |
|  |  | TC | Low altitude | 4.8241 | 0.2382 | A |
|  |  | TC | High altitude | 5.2183 | 0.7612 | A |
|  | gga_miR_1388a_3p | PXC | Low altitude | 7.7397 | 0.7600 | AB |
|  |  | PXC | High altitude | 5.2599 | 0.3181 | B |
|  |  | TC | Low altitude | 7.8573 | 0.4632 | A |
|  |  | TC | High altitude | 6.5948 | 1.7361 | AB |
|  | gga_miR_1388b_5p | PXC | Low altitude | 8.1339 | 0.8606 | A |
|  |  | PXC | High altitude | 5.5392 | 0.7459 | A |
|  |  | TC | Low altitude | 8.0660 | 0.3985 | A |
|  |  | TC | High altitude | 6.8285 | 1.7569 | A |
|  | miR-10c-5p | PXC | Low altitude | -0.8953 | 0.9100 | B |
|  |  | PXC | High altitude | 2.6000 | 2.3204 | AB |
|  |  | TC | Low altitude | 2.1557 | 1.6656 | AB |
|  |  | TC | High altitude | 3.5632 | 0.5380 | A |

**Table S4.** Number of Differentially Expressed Genes and Enrichment of Their Pathways in 48 Samples

| **Comparison type** | | **Tissue** | **Number of**  **DEGs** | **Functional category** | | | | **Total**  **pathway** | **Number of Up-regulated DEGs** | **Number of Down-regulated DEGs** |
| --- | --- | --- | --- | --- | --- | --- | --- | --- | --- | --- |
|  |  |  |  | **GO-BP** | **GO-MF** | **INTERPRO** | **KEGG** |  |  |  |
| T-test | TC vs LC | Brain | 196 | 19 | 1 | 14 | 1 | 35 | 175 | 21 |
|  |  | Heart | 18 | 0 | 0 | 0 | 0 | 0 | 8 | 10 |
|  |  | Lung | 137 | 7 | 3 | 7 | 2 | 19 | 39 | 98 |
|  |  | Liver | 1017 | 21 | 17 | 46 | 6 | 90 | 169 | 848 |
|  | HLC vs LTC | Brain | 62 | 0 | 0 | 0 | 0 | 0 | 34 | 28 |
|  |  | Heart | 209 | 10 | 0 | 6 | 2 | 18 | 88 | 121 |
|  |  | Lung | 467 | 30 | 8 | 16 | 3 | 57 | 150 | 317 |
|  |  | Liver | 425 | 9 | 4 | 14 | 12 | 39 | 138 | 287 |
|  | TC vs LTC | Brain | 107 | 3 | 1 | 2 | 2 | 8 | 84 | 23 |
|  |  | Heart | 130 | 2 | 2 | 0 | 0 | 4 | 13 | 117 |
|  |  | Lung | 387 | 11 | 3 | 17 | 0 | 31 | 127 | 260 |
|  |  | Liver | 1176 | 16 | 12 | 32 | 8 | 68 | 192 | 984 |
|  | HLC vs LC | Brain | 91 | 3 | 1 | 3 | 1 | 8 | 65 | 26 |
|  |  | Heart | 105 | 2 | 0 | 0 | 1 | 3 | 78 | 27 |
|  |  | Lung | 185 | 10 | 3 | 8 | 2 | 23 | 65 | 120 |
|  |  | Liver | 377 | 7 | 0 | 14 | 10 | 31 | 104 | 273 |
|  | TC vs HLC | Brain | 47 | 1 | 0 | 1 | 0 | 2 | 25 | 22 |
|  |  | Heart | 231 | 3 | 3 | 2 | 4 | 12 | 54 | 177 |
|  |  | Lung | 107 | 3 | 0 | 4 | 0 | 7 | 53 | 54 |
|  |  | Liver | 421 | 10 | 6 | 12 | 2 | 30 | 98 | 323 |
|  | LTC vs LC | Brain | 61 | 1 | 1 | 3 | 0 | 5 | 31 | 30 |
|  |  | Heart | 68 | 0 | 0 | 0 | 0 | 0 | 53 | 15 |
|  |  | Lung | 122 | 3 | 0 | 1 | 0 | 4 | 77 | 45 |
|  |  | Liver | 109 | 5 | 0 | 1 | 0 | 6 | 62 | 47 |

**Table S5.** Distribution of SNPs.

| Category |  | Number |
| --- | --- | --- |
| intronic |  | 5,055,796 |
| intergenic |  | 3,981,930 |
| ncRNA_intronic | | 1,030,664 |
| upstream |  | 182,660 |
| downstream | | 178,692 |
| exonic | synonymous | 104,003 |
|  | nonsynonymous | 46,306 |
|  | stopgain | 504 |
|  | stoploss | 83 |
|  | unknown | 1 |
| ncRNA_exonic | | 106,971 |
| UTR3 |  | 89,201 |
| UTR5 |  | 25,684 |
| splicing |  | 17,091 |
| Total | | 10,819,586 |
